# Influence of Obturation Technique on the Retreatability and Push-Out Bond Strength of Bioceramic Sealers: An In Vitro SEM Study

**DOI:** 10.3390/ma19143018

**Published:** 2026-07-13

**Authors:** Wissam Sami Haichal, Edoardo Ferrari Cagidiaco, Sara Alfonso, Giulia Verniani, Denise Irene Karin Pontoriero

**Affiliations:** 1 Department of Medical Biotechnologies, Faculty of Dentistry, University of Siena, 53100 Siena, Italy; saaraalfonso@gmail.com (S.A.); giuliaverniani96@gmail.com (G.V.); denisepontoriero@yahoo.it (D.I.K.P.); 2 University Hospital ‘Policlinico Santa Maria alle Scotte’, University of Siena, Viale Bracci 14, 53100 Siena, Italy; 3 Department of Prosthodontics and Dental Materials, University of Siena, 53100 Siena, Italy; edoardo.ferrari.cagidiaco@gmail.com

**Keywords:** endodontic retreatment, bioceramic sealer, warm vertical compaction, single-cone technique, push-out bond strength, scanning electron microscopy

## Abstract

Bioceramic sealers have gained widespread acceptance in contemporary endodontics due to their favorable biological properties, dimensional stability, and bioactivity. However, their strong adhesion to dentinal walls and penetration into dentinal tubules may complicate removal during endodontic retreatment procedures. Additionally, the obturation technique may influence both the retreatability of root canal filling materials and the bond strength of sealers and resins to radicular dentin. Methods: One hundred extracted human single-rooted teeth were instrumented using the WaveOne Gold system and obturated with a bioceramic sealer using two techniques: single-cone (Group A, *n* = 50) and warm vertical compaction (Group B, *n* = 50). After sealer setting, retreatment procedures were performed using ProTaper Universal Retreatment files combined with passive ultrasonic irrigation. Scanning electron microscopy was used to evaluate smear layer removal and dentinal tubule obstruction. Eighty specimens were subsequently re-obturated with a bioceramic sealer and divided into four subgroups according to the obturation technique used before and after retreatment. A push-out bond strength test was performed on coronal, middle, and apical root sections. Statistical analysis was conducted using SPSS software with a significance level set at *p* ≤ 0.05. Results: No statistically significant differences were observed between the groups regarding the working time required for root filling removal (*p* > 0.05). However, significant differences were found in push-out bond strength values among the experimental groups (*p* < 0.05). Specimens obturated using warm vertical compaction showed significantly higher bond strength compared with the single-cone technique. Scanning electron microscopy observations revealed less residual bioceramic sealer on dentinal walls in the warm vertical compaction group. Conclusions: Within the limitations of this in vitro study, the warm vertical compaction technique demonstrated superior bonding performance and resulted in less residual bioceramic sealer after retreatment compared with the single-cone technique.

## 1. Introduction

The primary objective of endodontic treatment is the chemo-mechanical debridement of the root canal system and the removal of infected or necrotic tissues to allow proper shaping and obturation of the root canal space [1,2]. Failure rates of primary endodontic treatment have been reported to range between 15% and 32% [3]. When failure occurs, nonsurgical endodontic retreatment is usually considered the treatment of choice.

However, retreatment procedures are technically demanding because complete removal of previous root canal filling materials is often difficult to achieve [4]. Residual obturation materials may act as a physical barrier that prevents irrigants from reaching microorganisms located in inaccessible areas of the root canal system such as dentinal tubules, lateral canals, and isthmuses [5]. Furthermore, residual materials may compromise the adhesion of new filling materials to radicular dentin [6].

Bioceramic sealers have gained increasing attention in endodontics because of their favorable physicochemical and biological properties, including biocompatibility, bioactivity, dimensional stability, and excellent sealing ability [7]. These materials are mainly composed of calcium silicates, calcium phosphate, calcium hydroxide, and zirconium oxide as a radiopacifier. Their small particle size and high flowability allow penetration into dentinal tubules and irregularities of root dentin [8,9].

Despite these advantages, the strong adhesion of bioceramic sealers to dentin and their ability to penetrate dentinal tubules may make their removal during retreatment procedures more difficult [9]. Several factors may influence the efficiency of sealer removal, including obturation technique, irrigation protocols, instrumentation systems, and the physicochemical properties of the sealer [10].

Different techniques have been proposed to improve removal of root canal filling materials during retreatment, including rotary or reciprocating instrumentation systems and activation methods such as passive ultrasonic irrigation [11]. Ultrasonic activation enhances irrigant penetration and facilitates debris removal through acoustic streaming and cavitation effects [12].

Therefore, the aim of this study was to evaluate the influence of different obturation techniques using bioceramic sealers on the efficiency of sealer removal and on push-out bond strength after endodontic retreatment.

The first null hypothesis tested was that no significant difference would exist between the single-cone and warm vertical compaction techniques regarding the bond strength of retreated teeth and the second null hypothesis that no significant difference would exist between the single-cone and warm vertical compaction techniques regarding residual sealer after retreatment.

## 2. Materials and Methods

### 2.1. Specimen Selection

One hundred extracted human single-rooted teeth were selected for this study. Teeth presenting caries, fractures, previous endodontic treatment, root resorption, or curvature greater than 20° were excluded.

Soft tissue remnants were removed using hand scalers, and the teeth were stored in physiological saline until use.

### 2.2. Root Canal Preparation

The specimens’ crowns were sectioned at the cementum–enamel junction. Working length was determined using a size 10 K-file inserted until visible at the apical foramen and then reduced by 1 mm.

Root canal preparation was performed using the WaveOne Gold system (Dentsply Maillefer, Ballaigues, Switzerland).

The final irrigation protocol included: 5% sodium hypochlorite for 5 min and 17% EDTA for 1 min.

Passive ultrasonic irrigation was used to activate irrigating solutions, followed by a final rinse with saline solution.

### 2.3. Root Canal Obturation

Specimens were randomly divided into two groups according to obturation technique: Group A (n = 50): Single-cone technique, wherein a single gutta-percha point, shaped to match the canal’s final configuration and taper, is inserted into the canal to achieve complete obturation without accessory points, and has been associated with favorable clinical outcomes in round, narrow, and anatomically simple root canals. A WaveOne Gold (Dentsply Maillefer, Ballaigues, Switzerland) gutta-percha cone, sized to match the final prepared canal, was inserted into the canal with bioceramic sealer Ceraseal (Meta Biomed Co., Ltd., Cheongju, Republic of Korea) to the working length. The excess gutta-percha was removed at the canal orifice level with a heat carrier (Obtura, Meta systems EQ-V, Algonquin, IL, USA), and the remaining gutta-percha was compacted using pluggers (Hu-Friedy, Chicago, IL, USA) [13].

Group B (n = 50): Warm vertical compaction technique, in which obturation was performed with a gutta-percha cone. A 0.5 mm tip cone was trimmed, coated with a bioceramic sealer, and placed into the root canal 0.5 mm short of the working length. The cone was condensed and compacted 5 mm from the apex (down packing). Roots were then back-filled with thermoplastic injectable gutta-percha. This device was preset to 180 °C for apical compaction of gutta-percha, as recommended by the manufacturer. Vertical condensation was then performed using calibrated pluggers (RCP #8-1/2 and #9-1/2) [14].

Specimens were incubated at 37 °C and 100% humidity for two weeks to allow complete sealer setting [10].

### 2.4. Retreatment Procedures

After two weeks, the specimens of Groups A and B were then retreated. Root canal fillings were removed using ProTaper Universal Retreatment files (Dentsply Maillefer, Ballaigues, Switzerland), and the working time needed for each specimen and the media of the working times of the one hundred specimens (in minutes and seconds) were collected, and the data were statistically analyzed by SPSS software (version 25). The primary benefit of ProTaper Universal Retreatment files is their ability to remove dentin and debris in a coronal direction, hence minimizing the extrusion of filling material through the apical foramen, which has been proven to be effective and safe [15].

The working time needed for retreatment was taken and then statistically evaluated to observe if longer working time was spent to remove bioceramic sealer from root canal when single cone instead than warm condensation procedure.

### 2.5. Second Obturation

The remaining 80 specimens were re-obturated using a bioceramic sealer and divided into four subgroups of 20 specimens each. Accordingly, the 1st and 2nd type of endodontic obturation were made: SubGroup 1: Single cone → Single cone; SubGroup 2: Single cone → Warm vertical compaction; SubGroup 3: Warm vertical compaction → Warm vertical compaction; SubGroup 4: Warm vertical compaction → Single cone.

The obturation procedures were performed as already described. After being re-obturated, the specimens were kept in a wet environment for two weeks before testing (Table 1).

### 2.6. SEM Evaluation of Sealer Debris

SEM observations were made on 40 specimens, with 20 specimens from Group A and B after being retreated for the second time.

Irrigation was performed with 5% sodium hypochlorite for 5 min and 17% EDTA for 1 min. Passive ultrasonic irrigation (PUI) was used to enhance removal of debris and the smear layer, followed by the final rinse with saline solution. Dedicated paper points were used to dry the canals. Then, the dental specimens were partially cut longitudinally by using a low-speed saw (Isomet, Buehler, New York, NY, USA) with cooling water and finally fractured. All specimens were examined using a scanning electron microscope (JSM-6060LV, Jeol Ltd., Tokyo, Japan) at various magnifications after being placed on matrices and gold-sputtered using an EMITECH K550 sputtering equipment (Quorum Technologies Ltd., East Grinstead, UK).

The amount of smear layer was evaluated based on SEM micrographs obtained at ×1000 magnification, using a rating scale from 1 to 5. Three scanning shots were made randomly along the root canal, in the coronal, medium and apical area.

1: Greater than or equal to 50% of the surface covered by the smear layer.
2: Less or equal to 40% of the surface covered by the smear layer.
3: Less or equal to 30% of the surface covered by the smear layer.
4: Less or equal to 20% of the surface covered by the smear layer.
5: Less or equal to 10% of the surface covered by the smear layer.

SEM images were analyzed independently by two calibrated examiners. In cases of disagreement, a joint re-evaluation was performed until consensus was reached. Collected data were analyzed through a statistical test (Wilcoxon and Fisher), setting the *p*-value at *p* < 0.01.

### 2.7. Push-Out Bond Strength Test

The 60 specimens were divided into four subgroups of fifteen specimens each. Roots were embedded in acrylic resin (Ivoclar Vivadent, Schaan, Liechtenstein) and sectioned perpendicular to their long axis into 2 mm-thick slices representing coronal, middle, and apical thirds, using a low-speed saw, under cooling water. Four–five slides were obtained from each sample root. The thickness of each slice was measured with a stainless-steel hardened digital caliper of 0.1 mm accuracy (ikkegol, Shenzhen, China). Push-out bond strength was measured using a universal testing machine at a crosshead speed of 0.5 mm/min.

The root filling in each specimen was subjected to a compressive load using stainless-steel cylindrical plungers of different diameters (1 mm for coronal specimens, 0.7 mm for middle specimens, 0.5 mm for apical specimens), so as to touch only the endodontic obturation without stressing the surrounding root canal walls. The plunger was mounted on the upper part of a universal testing machine (Triax 50, Controls SPA, Milano, Italy). The specimens were aligned over a support jig in an apical-to-coronal direction to avoid any constriction interference. The tests were conducted at a cross-head speed of 0.5 mm/min using a 500 N load cell. The interfacial area was calculated for each specimen according to the following equation [16]:

Push-out bond strength (MPa) = Maximum load/Adhesion surface area

The adhesion surface area was calculated according to the following formula:

The interfacial area was calculated as the lateral surface of a truncated cone using the formula A = π(R + r)[h^2^ + (R − r)^2^]^0.5^, where π = 3.14, R is the coronal post radius, r is the apical post radius, and h is the thickness of the slice.

The Kruskal–Wallis statistical test was used with *p* = 0.001.

## 3. Results

### 3.1. Working Time of the Retreatments

Regarding the working time of retreatment, the results of this study showed that there were no differences between the two tested obturation techniques and no statistically significant difference was observed between groups (*p* > 0.05) (Table 2). Mean working time was 153.68 ± 60.72 for group SC versus 150.80 ± 57.24 for group WVC.

This can be explained by the working efficiency of the rotary instruments specifically used to retreat the specimens.

### 3.2. Amount of Sealer Debris

**Figure 1:**
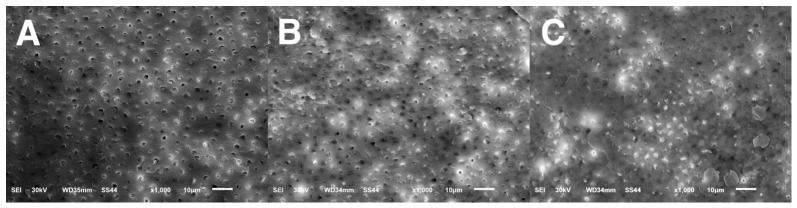
Microphotographs show an example of a specimen with a score of 3; it is possible to note tubules still close at magnification (×1000) after not being cleaned from endodontic obturation; (**A**) coronal part, (**B**) middle part, (**C**) apical part.

**Figure 2:**
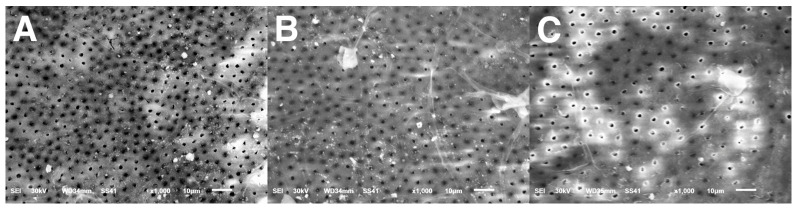
Microphotographs show an example the specimen with a score of 5; it is possible to note open tubules at magnification (×1000) after being well-cleaned from endodontic obturation; (**A**) coronal part, (**B**) middle part, (**C**) apical part.

**Figure 3:**
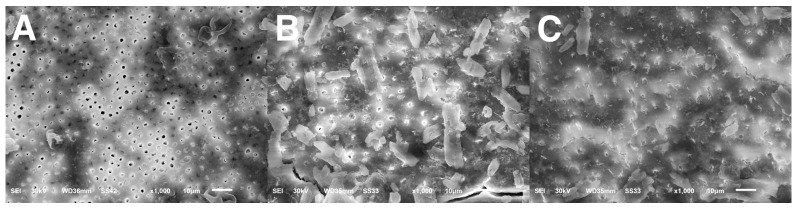
Microphotographs show an example of a specimen with a score of 1; it is possible to note tubules still close and remnant cement covering the wide part of the dentinal wall at magnification (×1000); (**A**) coronal part, (**B**) middle part, (**C**) apical part.

Scanning electron microscopy analysis demonstrated that specimens initially obturated using warm vertical compaction showed less residual bioceramic sealer on dentinal walls after retreatment compared with specimens obturated using the single-cone technique (Figure 1, Figure 2 and Figure 3).

Data were analyzed using SPSS software (version 25) (SPSS Inc., PASW statistics for version 25, Chicago, Illinois, USA). Normal distribution was evaluated using the Kolmogorov–Smirnov test. Comparisons between groups were performed using Student’s *t*-tests and Kruskal–Wallis tests where appropriate. The significance level was set at *p* ≤ 0.05 (Table 3).

### 3.3. Push-Out Bond Strength Test

The push-out test showed that SubGroup 1, where the SC obturation was made twice, was the worst, while SubGroup 3, where the WVC was made twice, had the best results. Median push-out value was higher among SubGroup 3, followed by SubGroup 4, then SubGroup 2, and was the least for SubGroup 1. In SubGroups 3 and 4, the first obturation technique was the WVC, while in SubGroups 1 and 2 it was SC. (Table 4).

Regarding the statistical analysis of push-out bond strength values, significant differences were observed between SubGroup 1 and the other experimental SubGroups (*p* = 0.001). No differences were found among SubGroups 2, 3 and 4.

Pairwise comparison between different groups demonstrates a statistically significant difference between group 1 versus 2 (*p* = 0.001), between SubGroup 1 versus 3 (P2 = 0.015 *) and between SubGroups 1 versus 4 (*p* = 0.002). No statistically significant difference was detected between SubGroups 2 versus SubGroups 3 (*p* = 0.275), between SubGroups 2 versus 4 (*p* = 0.658), and between SubGroups 3 versus SubGroups 4 (*p* = 0.513).

## 4. Discussion

Endodontic retreatment aims to eliminate microorganisms and residual filling materials from previously treated root canals to allow effective disinfection and successful re-obturation [17]. The present study demonstrated that the WVC technique resulted in significantly higher push-out bond strength values compared with the single-cone technique when the roots were retreated. Therefore, the first null hypothesis was rejected.

SEM observations revealed that specimens obturated with the warm vertical compaction technique exhibited less residual sealer after retreatment. The thicker sealer layer typically associated with the single-cone technique may explain the increased presence of sealer remnants during retreatment procedures [18,19]. For this reason, the second null hypothesis tested was rejected.

It was interesting to correlate the push-out results and SEM observations; from this correlation it may be concluded that the possibility of better removing the bioceramic sealer from the root canal walls may increase the bond strength of the second obturation when done with WVC technique.

During the retreatment of the samples, passive ultrasonic irrigation was used to enhance smear layer removal and improve irrigant penetration into the complex anatomy of the root canal system and to better clean the root canal space. Previous studies have demonstrated that ultrasonic activation significantly improves removal of the smear layer and sealer remnants compared with conventional irrigation techniques [20].

In this study, the coronal and middle thirds of the root canal, ideal areas for an adhesive restoration after the retreatment, showed an improved removal of bioceramic sealer when warm vertical compaction was originally used. This is probably because the ultrasonic activation with saline enhances cleaning efficiency [21]. Similar observations were reported by Pontoriero et al., who highlighted the importance of sealer properties and dentin interaction in the long-term outcome of endodontic treatments [14]. The high solubility of the bioceramic sealer before setting is the result of hydrophilic nanosized particles being present in the bioceramic sealer, which increases their surface area and allows more liquid molecules to come into contact with the sealer [22].

The working time of retreatments and their results showing that there were no differences between the two tested obturation techniques can be due to the efficiency of the rotary instruments specifically used to retreat the specimens.

This study has several limitations. First, the study was conducted under in vitro conditions, which may not fully reproduce clinical situations. Second, anatomical variability among teeth may influence dentinal tubule distribution and sealer penetration [23,24]. Third, SEM analysis and all endodontic procedures may involve some degree of operator interpretation.

Further studies are needed to evaluate alternative retreatment protocols and activation techniques to improve the removal of bioceramic sealers and enhance retreatment outcomes.

## 5. Conclusions

Within the limitations of this in vitro study, the following conclusions can be drawn:

1. No difference in the working time required to remove root canal fillings between the two obturation techniques was found when single-cone or warm vertical compaction were originally used.
2. Warm vertical compaction showed significantly higher push-out bond strength than the single-cone technique when the bond strength was tested after retreatment.
3. Warm vertical compaction resulted in less residual bioceramic sealer after retreatment than the single-cone technique.

## Figures and Tables

**Table 1:** Description of subgroup composition related to the first and second type of obturation (legend: SC—Single cone; WVC—Warm vertical compaction).

Subgroups	1st Obturation	2nd Obturation
SubGroup 1	SC	SC
SubGroup 2	SC	WVC
SubGroup 3	WVC	WVC
SubGroup 4	WVC	SC

**Table 2:** Illustrates no statistically significant difference between studied groups in mean working time. Legend: SC, single-cone technique; WVC, warm vertical compaction. Data are presented as mean ± SD. Student’s *t*-test was used for statistical analysis.

	Group SC N = 50	Group WVC N = 50	Test of Significance
Working time (minutes and seconds) Mean ± SD	153.68 ± 60.72	150.80 ± 57.24	t = 0.268 *p* = 0.789

**Table 3:** The mean score of the remaining debris.

Groups	SubGroups	Scores
Group A (WVC)	SubGroup 1	1.23
	SubGroup 2	1.16
Group B (SC)	SubGroup 3	4.06
	SubGroup 4	3.03

**Table 4:** Global push-out comparison between studied groups.

	SubGroup 1 SC–SC	SubGroup 2 SC–CW	SubGroup 3 CW–CW	SubGroup 4 CW–SC	Test of Significance	Within Group Significance
Push out Median (min-max)	0.12 (0.01–0.99)	5.11 (1.42–54.07)	6.36 (1.14–10.42)	5.56 (1.51–11.9)	Kw = 41.32 *p* = 0.001 *	P1 = 0.001 * P2 = 0.015 * P3 = 0.002 * P4 = 0.275 P5 = 0.658 P6 = 0.513

P1: difference between group 1 vs. 2, P2: difference between group 1 vs. 3, P3: difference between group 1 vs. 4, P4: difference between group 2 vs. 3, P5: difference between group 2 vs. 4, P6: difference between group 3 vs. 4, Kw: Kruskal–Wallis test, * statistically significant.

## Data Availability

The data presented in this study are available on request from the corresponding author due to the data cannot be shared at this time because they are part of an ongoing research project and are reserved for planned analyses that have not yet been completed. Public release before these analyses are finalized could com-promise the integrity of the study and the novelty of future publications.
